# Supplementary material for: Ultrafast laser inscribed waveguides in tailored fluoride glasses: an enabling technology for mid-infrared integrated photonics devices
Source: Sci Rep. 2022 Aug 29;12:14674. doi: 10.1038/s41598-022-18701-y (PMC9424227; doi:10.1038/s41598-022-18701-y)
Supplement: Supplementary file 1 — Supplementary Information. [file 41598_2022_18701_MOESM1_ESM.pdf]

Ultrafast laser inscribed waveguides in tailored fluoride glasses: An enabling technology for mid-infrared integrated photonics devices : Supplementary document

T Toney Fernandez[1,*], B Johnston[1], S Gross[1], S Cozic[2], M Poulain[2], H Mahmodi[3], I Kabakova[3], M Withford[1], and A Fuerbach[1]

[1]MQ Photonics Research Centre, School of Mathematical and Physical Sciences, Macquarie University, NSW, 2109, Australia
[2]Le Verre Fluoré, 1 rue Gabriel Voisin - Campus KerLann, F-35170 Bruz, Brittany, France
[3]School of Mathematical and Physical Sciences, University of Technology Sydney, Ultimo, NSW, 2007, Australia
*Corresponding author: toney.teddyfernandez@mq.edu.au

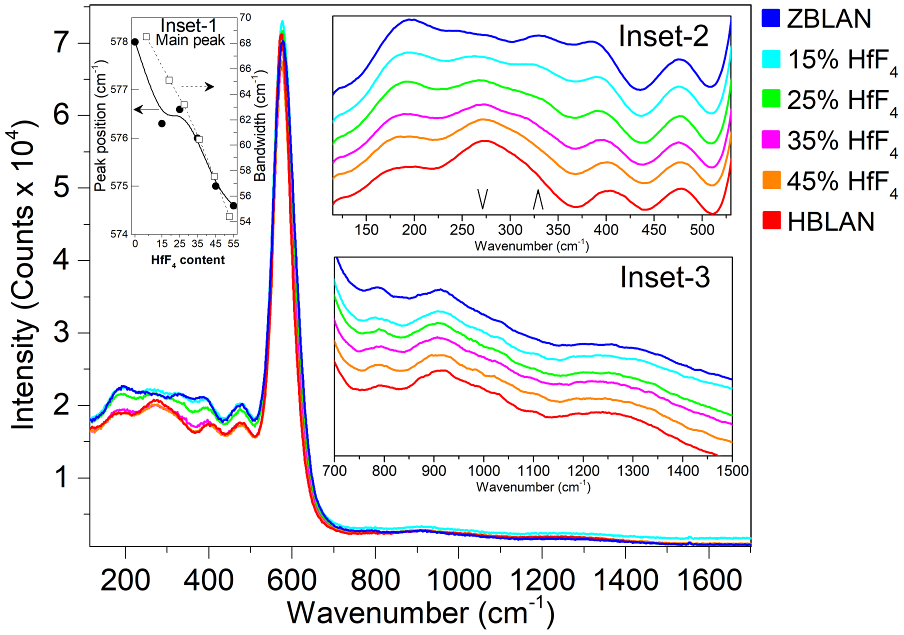

Fig. S1: Raman spectra of all six custom fluoride glasses. Inset-1: Main peak position and bandwidth variation across all the samples. Inset-2: Lower frequency peaks and Inset-3: higher frequency peaks respectively for all samples (vertically shifted for visibility)

Raman spectra of the glasses, acquired with 633 nm laser excitation, as shown in Fig. S1 has its distinctive peak vibrational frequency around 578 cm^{-1} for pure ZBLAN glass which monotonically decrease to 574.6 cm^{-1} for HBLAN as the hafnium content in the glass is raised to maximum (Inset-1). This main peak is specifically associated to the symmetric stretching vibration of terminal/non-bridging flouride bonds (F_{NBF}) attached to Zr or Hf atoms. It is intutive to associate this frequency reduction to the heavier mass of Hf, but this is not the case mainly because the vibration is exclusive

to the symmetric stretching of terminal/non-bridging flouride bonds due to the large mass discrepancy between zirconium/hafnium and flourine atoms. Also plotting the asymmetric stretching vibration of $F_N BF$, the frequency was found to increase from 388 cm^{-1} for pure ZBLAN to 404 cm^{-1} as the Hf content is increased to maximum. Zr- $F_N BF$ and Hf- $F_N BF$ are isomorphic and their bondings have similar force constant (376.6 & 377 Nm^{-1}), length (2.08 & 2.09 Å) and expected to have same coordination numbers. Hence substitution of Zr with Hf was not reported to have any effect on the symmetric stretching of terminal bonds. To explain the variation seen in our case, the effect of counter cations in the matrix should be taken into account. Ba, La, Al and Na are the counter cations in the matrix since Hf and Zr constitute the glass forming cations. Presence of a counter cation that can compete for terminal fluorides can vary the vibrational frequency of terminal fluoride vibrations of the glass matrix. Since the stoichiometry of counter cations are maintained a constant especially Al, reduction of Zr content reduces the Zr to Al ratio. Aluminium (11.3 Å) has a polarizability lower than Zr (170.6 Å) and higher than Hf (4.3 Å), hence reduction of Zr with increase in Hf reduces the vibrational frequency of F_{NBF} due to a higher localization of electrons within low polarizable Hf and Al sites. This is well reflected in (1) the large jump in main peak vibration for ZBLAN compared to the other Hf containing compositions (2) reduction of peak bandwidth (69→ 54 cm^{-1}) when moving towards a pure HBLAN composition. The vibration found at 475 and 400 cm^{-1} is associated to the asymmetric stretching vibrations of bridging and non-bridging flouride bonds respectively. The vibration at 273 cm^{-1} is predominantly observed only when Hf is present in the sample, whereas 330 cm^{-1} when Zr is present. Inset-2 is prepared to demonstrate this by deliberately offsetting the y-axis starting from pure HBLAN, ZrF_4 content is increasing for the the graph lying above it. The vibration intensity of both 273 cm^{-1} and 330 cm^{-1} peaks are seen increasing proportional to its respective glass former content indicating its active role. Both peaks are marked with a downward and upward arrow near the x-axis of inset-2 respectively. Hence this low frequency vibration could be attributed to the skeletal bending vibration with an active participation from respective glass former giving rise to two different vibration frequencies. Inset-3 highlights the higher lying frequencies that originates from the oxygen inclusions within the glass. They usually occupy a bridging position between two Zr or Hf due to their 2^- charge.
